# Ivermectin Treatment in Patients With Onchocerciasis-Associated Epilepsy: Protocol of a Randomized Clinical Trial

**DOI:** 10.2196/resprot.7186

**Published:** 2017-08-30

**Authors:** Robert Colebunders, Michel Mandro, Deby Mukendi, Housseini Dolo, Patrick Suykerbuyk, Marieke Van Oijen

**Affiliations:** ^1^ University of Antwerp Global Health Institute Antwerp Belgium; ^2^ Provincial Ministry of Health Bunia The Democratic Republic Of The Congo; ^3^ Centre Neuro Psycho Pathologique University of Kinshasa Kinshasa The Democratic Republic Of The Congo

**Keywords:** onchocerciasis, epilepsy, nodding syndrome, ivermectin, randomized clinical trial, Democratic Republic of the Congo

## Abstract

**Background:**

Many studies have reported an association between epilepsy, nodding syndrome (NS), and onchocerciasis (river blindness). A high prevalence of epilepsy has been noted particularly in onchocerciasis hyperendemic areas where onchocerciasis is not or insufficiently controlled with mass ivermectin distribution. There is evidence that increasing the coverage of ivermectin reduces the incidence of epilepsy, and anecdotal evidence suggests a reduction in seizure frequency in onchocerciasis-associated epilepsy (OAE) patients who receive ivermectin. Finding an alternative treatment for epilepsy in these patients will have major consequences.

**Objective:**

The goal of the study is to assess whether ivermectin treatment decreases the frequency of seizures and leads to seizure freedom in OAE patients, including patients with NS. If we are able to demonstrate such an effect, this would strengthen the argument that onchocerciasis is causing epilepsy and therefore we should increase our efforts to eliminate onchocerciasis.

**Methods:**

We will conduct a randomized clinical trial in the Democratic Republic of Congo to compare seizure freedom in onchocerciasis-infested epilepsy patients who receive immediate ivermectin treatment with delayed (after 4 months) ivermectin treatment. All participants will simultaneously receive antiepilepsy drugs (AEDs) according to local guidelines for epilepsy treatment. The primary endpoint is seizure freedom defined as no seizures during the 4 month of follow-up. Secondary endpoint is significant (>50%) seizure reduction compared to baseline seizure frequency. Reduction of seizures will be compared between ivermectin and nonivermectin arms.

**Results:**

Start of enrollment is planned for August 2017, and we expect to have enrolled all 110 participants by December 2017. Results are expected in June 2018.

**Conclusions:**

If ivermectin treatment in addition to AEDs is able to lead to seizure freedom or significantly reduces seizure frequency in OAE patients, this will have major consequences for epilepsy treatment in onchocerciasis-endemic regions. Ivermectin is donated for free and in non Loa-Loa–endemic regions has negligible side effects. Reducing the burden of epilepsy will have a major impact on quality of life and socioeconomic status of families with affected members in Africa.

**Trial Registration:**

ClinicalTrials.gov NCT03052998; https://clinicaltrials.gov/ct2/show/NCT03052998 (Archived by WebCite at http://www.webcitation.org/6roFVQSG0)

## Introduction

### Onchocerciasis-Associated Epilepsy

Many studies have reported an association between epilepsy, nodding syndrome (NS), and onchocerciasis [1-4]. A meta-analysis of African population-based surveys showed a variation in epilepsy prevalence consistent with onchocerciasis prevalence, with epilepsy prevalence being increased, on average, by 0.4% for each 10% increase in onchocerciasis prevalence [2]. NS is an epileptic disorder occurring in children in onchocerciasis (river blindness) endemic regions, initially only observed in South Sudan, Uganda, and Tanzania [5]. NS occurs in previously healthy children, aged mainly from 5 to 18 years and is characterized by head-nodding, an atonic epileptic seizure [5,6]. Individuals may also develop other types of seizures and stunted growth. Many affected children not only suffer from recurrent seizures but also from severe cognitive, behavioral, and psychiatric problems [7,8]. NS should be considered part of a spectrum of onchocerciasis-associated epilepsy (OAE) disorders [9]. We recently suggested that these epileptic disorders share etiological factors related to *Onchocerca volvulus* infection and therefore considered ivermectin, used to treat onchocerciasis, as a treatment option for OAE [10].

### Ivermectin Treatment May Prevent Onchocerciasis-Associated Epilepsy

Ivermectin is effective against many types of parasites and is recommended every 6 months for treatment of onchocerciasis. Medication is taken orally and generally well tolerated [11]. Accumulating evidence is suggesting that ivermectin may prevent OAE. After the introduction of biannual mass treatment with ivermectin in 2012, no new NS cases have been observed in Northern Uganda [12]. In a recent age-matched case control study in Titule, Bas Uélé, in the Democratic Republic of Congo (DRC), 16/18 (89%) of patients with epilepsy had not taken ivermectin the year before they developed epilepsy, compared to 7/18 (39%) controls (*P*=.002) [13]. We recently visited several villages in the Mbam valley (an onchocerciasis-endemic area in Cameroon) where the prevalence of epilepsy in 1992 ranged between 4% and 8% and most epilepsy cases occurred in people aged younger than 20 years [3]. In 2015, after more than 15 years of administering ivermectin annually, nearly all epilepsy cases were in people over the age of 20 years, also suggesting a protective effect of ivermectin on the incidence of epilepsy (R Colebunders, personal communication). A similar age shift of epilepsy cases to older age groups after the introduction of ivermectin was reported in an onchocerciasis-endemic region in Nigeria [14]. With ivermectin distribution only once a year, it may take many years with high treatment coverage before a significant effect on the incidence of OAE is observed. Indeed, several months after the administration of one dose of ivermectin, microfilariae reappear and therefore create a risk for the infected individual to develop epilepsy. For this reason, in order to decrease the incidence of OAE more rapidly, at least biannual intake of ivermectin may be required.

### Pathophysiological Mechanism of Onchocerciasis-Associated Epilepsy

The pathophysiological mechanism of OAE remains unclear. In recent studies, *O volvulus* DNA was never isolated from cerebrospinal fluid (CSF) in patients with NS [15]. However, several of the patients enrolled in these studies had taken ivermectin in the past. In 1938, before the use of ivermectin, microfilariae in CSF were described by Casis Sacre [16] in Mexican patients with onchocerciasis with NS and Nakalanga-like clinical manifestations. Dead and live microfilariae were also found in 1959 by Mazotti [17] in CSF of patients with onchocerciasis treated with diethylcarbamazepine. In 1976, Duke et al [18] noted the presence of small numbers of *O volvulus* microfilariae in the CSF (<2 mf/mL) in 5 of 8 untreated heavily infected (>100 mf/mg skin) onchocerciasis patients. During diethylcarbamazine treatment, in 10 out of 11 heavily infected patients presenting with an ocular form of onchocerciasis, the numbers of *O volvulus* microfilariae in the CSF increased even up to 8 to 31 mf/mL [18]. Patients with *O volvulus* infection receiving diethylcarbamazine were reported to develop optic atrophy, probably because of a Mazotti reaction caused by dead microfilariae present in the optic nerve [19]. Perhaps this nerve could be an entry point of the microfilariae to the brain. Another explanation for the association between onchocerciasis and epilepsy could be the occurrence of an autoimmune response related to the *O volvulus* infection [20] leading to seizures.

Ivermectin acts on the chloride-dependent channels of both glutamate and γ-aminobutyric acid, interrupting neurotransmission in invertebrates. However, in humans, several mechanisms of brain protection exist, including P-glycoprotein, present on the apical face of endothelial cells of the blood-brain barrier and coded by the MDR1 gene [21]. Therefore in humans ivermectin generally does not enter the brain [21] and would not be able to kill microfilariae inside the brain. Ivermectin could, however, reduce the microfilariae load outside the brain and either reduce the risk that additional microfilariae penetrate the brain or reduce the neurotoxic immunological response caused by the microfilarae.

One dose of ivermectin eliminates microfilarae very rapidly [22]. A mathematical model predicted that microfilaridermia would be reduced by half 24 hours after the intake of ivermectin [23]. Therefore, if the microfilariae load plays an important role in causing OAE, it may be that ivermectin also has a rapid effect on the frequency of seizures.

### Ivermectin Treatment May Decrease Seizure Frequency in Patients With Onchocerciasis-Associated Epilepsy

In a study in Kabarole district in Uganda in 1992, 34/91 (37%) patients reported some decrease in either the frequency or severity of seizures after 1 dose of ivermectin (150 µg/kg) [24]. After being treated with ivermectin, 13/91 (14%) individuals had no seizures for 3.7 months (on average). Seizures were unchanged in 51/91 (56%), and worsened in 6/91 (7%) [24]. In a recent trial in the DRC comparing moxidectin (an anti- *O volvulus* experimental drug with a longer half-life than ivermectin) with ivermectin, 6 (80%) out of 7 *O volvulus*-infested patients with epilepsy became seizure free after treatment with moxidectin or ivermectin (the randomization code has not been broken yet). In one person, seizure frequency was significantly reduced over the 18-month follow-up period. In this person, microfilariae remained present in skin snips, although at a lower level than before the onchocerciasis treatment. In all subjects who became seizure free, the skin snips too became microfilaria free [M Mandro, unpublished].

To assess whether ivermectin treatment may reduce the frequency of seizures and lead to seizure freedom, we intend to conduct a short proof-of-concept randomized clinical trial to compare immediate ivermectin treatment with delayed (after 4 months) ivermectin treatment in onchocerciasis-infested persons with epilepsy. The primary outcome is seizure freedom at 4 months. Reducing the burden of epilepsy will have a major impact on quality of life and socioeconomic status of families with affected members in Africa. If we are able to demonstrate an effect of ivermectin on the frequency of seizures, this would be an extra argument that onchocerciasis is causing epilepsy and that therefore we should increase our efforts to eliminate onchocerciasis.

## Methods

### Study Design

This is a proof-of-concept randomized treatment trial consisting of 2 treatment arms, immediate (arm A) and delayed (4 months later) ivermectin treatment (arm B). A computer-based, preplanned age and frequency of seizures stratified randomization list will be used. The trial will not be placebo-controlled as this will be costly and we do not expect this to greatly influence reporting of seizures, our primary outcome. Epilepsy patients in both arms will additionally receive antiepileptic drugs (AEDs) following local guidelines of epilepsy treatment in DRC. Study investigators collecting and analyzing data will be blinded for treatment.

### Study Population, Setting, and Enrollment

The trial will take place in selected villages in the Logo health zone, an onchocerciasis-endemic region in the Ituri province in the DRC, in areas where so far mass ivermectin administration has not been implemented but where the national onchocerciasis program is planning to start community-directed distribution of ivermectin in 2017. Pilot studies by our group in this area show prevalence estimates of OAE of approximately 5% [25].

Before starting, the village chief and community health workers will be informed of the purpose and specifics of our study. When permission is obtained, our medical team will visit the village and set up a mobile clinic. Patients who may fulfill eligibility criteria are selected and examined for a screening visit after informed consent is obtained from patient and/or caretaker in the local language (Alur). Patients who meet the enrollment criteria (see Textbox 1) are invited to participate in the treatment trial. Detailed information about the trial is given in local language, and enrollment informed consent is obtained.

Our study population consists of epilepsy patients aged 5 years and older with onchocerciasis and without other obvious risk factors for epilepsy.

### Trial Registration

The trial is registered at ClinicalTrials.gov [NCT03052998] and will be registered at the World Health Organization International Registry Network.

### Screening of Epilepsy Patients for Onchocerca volvulus Infection

After informed consent is obtained, trial candidates with epilepsy will be tested for the presence of antibodies to the parasite antigen OV16 with the SD BIOLINE Onchocerciasis IgG4 Rapid Test (Standard Diagnostics Inc). Also, a skin snip will be taken from the left and right iliac crests with a Holtz corneoscleral punch (2 mm). One sterilized punch will be used per subject. Each snip will be weighed on an analytical balance and incubated for 24 hours in isotonic saline in a well of a flat-bottomed microtiter plate. The microfilariae that emerge will be counted using an inverted microscope. The number of microfilariae in each well and the weight of the associated skin snip before incubation will be recorded. The mean of the skin microfilarial (mf) density (mf count/weight) of snip across all 4 skin snips will be calculated and recorded as mf/mg. Skin biopsies will then be stored in 90% ethanol to be tested for *O volvulus* by an in-house polymerase chain reaction method (supplemental methods).

### Diagnosis of Onchocerca volvulus Infection

Diagnosis of onchocerciasis infection will be made when microfilariae are detected in skin snip and/or antibodies to the parasite antigen OV16 are detected.

### Diagnosis of Epilepsy

To identify eligible epilepsy patients with generalized tonic clonic seizures, we will use a 10-item epilepsy questionnaire previously used in international epilepsy prevalence studies [26,27]. A person identified on the basis of this questionnaire as possibly a person with epilepsy will be examined by a neurologist to make an accurate diagnosis according to definitions proposed by the International League Against Epilepsy [28]. A case of epilepsy will be defined as a patient who has had (1) at least 2 times, unprovoked and without fever, lost consciousness with convulsions with a minimal time difference of 24 hours between the 2 events or (2) 1 unprovoked seizure and a probability of future seizures similar to the general recurrence risk after 2 unprovoked seizures. Detailed questions including the age at seizure onset, seizure frequency, and family history of seizures are part of the baseline questionnaire. The eligibility criteria are listed in Textbox 1.

**Selection criteria.**

Inclusion criteria:

- Age 5 years and older
- Signed informed consent form
- Normal neurological development until onset of epilepsy
- Onset of epilepsy between ages of 5 and 18 years
- Seizure frequency of ≥2 seizures per month
- Presence of microfilariae in skin snip and/or antibodies against OV16
- Generalized (primarily or secondarily) tonic clonic seizures are present

Exclusion criteria:

- Ivermectin intake in the last 9 months
- Pregnancy or breastfeeding
- Known or suspected allergy to ivermectin
- *Loa Loa* microfilariae in blood^a^
- Epilepsy with known cause (eg, severe head trauma, perinatal asphyxia, patients with a history of cerebral malaria, meningitis, or encephalitis)
- Concomitant acute illness or chronic medication use
- Chronic alcohol or substance use
- Use of antiepilepsy drugs in the past 2 weeks

^a^Treatment with ivermectin in persons with *Loa Loa* co-infection may cause fatal encephalopathy.

Blood samples will be tested for *Taenia solium* antibodies and antigen, but in the absence of a point-of-care test, results will only become available after screening. Therefore these test results are not part of the eligibility criteria. However, they will be taken into account in analyzing the results.

### Ivermectin Treatment Strategy

Following recommendations, 1 dose of 150 µg/kg ivermectin (Mectizan) will be administered orally, and treatment will be directly observed. Ivermectin is generally well tolerated. Common side effects of ivermectin include fever, itching, skin rash, edema, myalgia, and headache.

### Anti-Epileptic Drug Treatment Strategy

We designed a standard epilepsy treatment protocol for both treatment arms. To summarize, we will start with phenobarbital 100 mg once a day which may be increased to 150 to 200 mg per day after 2 months if there is insufficient seizure reduction (less than 50% reduction of seizure frequency). If there are contraindications for use of phenobarbital (intellectual or behavioral disorders) or persistent side effects, carbamazepine will be prescribed (in adults initial dose 100 to 200 mg per day, maintenance dose 400 to 1400 mg; in children initial dose is 5 mg/kg per day, maintenance dose 10 to 30 mg/kg per day). In the case of side effects related to carbamazepine, we will start with sodium valproate (in adults initial dose 400 mg per day, maintenance dose 400 to 2000 mg per day; in children initial dose 15 to 20 mg/kg per day and maintenance dose 15 to 30 mg/kg per day). In the case of carbamazepine and sodium valproate, we will prescribe initial dose in all patients and will increase this to the lowest maintenance dose at 2-week visit. In case of dose-determined side effects, dose reduction is permitted. Dose may be increased at the 2-month visit if there is insufficient seizure control (less than 50% reduction in seizure frequency). Individual treatment decisions will be made by the team physician who has received specific training in epilepsy treatment and can consult with the team neurologist. Patient and family will be informed of the following regarding epilepsy treatment [29]:

- Delay in onset of effect and the time course of treatment
- Potential side effects and the risk of these symptoms (seek help promptly if these are distressing)
- Risk of abrupt discontinuation/withdrawal symptoms on missing doses
- Need for regular follow-up

### Compliance

We will perform indirect (pill count) and direct (AED blood levels) methods to check for compliance to AED treatment. We will train community volunteers to assist the research team and local health team with the follow-up of the trial participants and for compliance monitoring at the home of the participants. The center visit at week 2 is scheduled to check for side effects in order to minimize withdrawal from AED treatment. Although noncompliant patients are expected to be equally distributed among treatment arms because of randomization and therefore not influence outcome, it is important to put effort into minimizing noncompliance, especially since we intend to continue treatment with AEDs past the duration of the trial regardless of the results. Community volunteers will also be trained to become community directed distributors of ivermectin after completion of the trial.

### Endpoints

Primary endpoint is proportion of patients who have achieved seizure freedom after 4 months. Seizure freedom will be defined as no seizures the last month of the trial (month 4). Secondary endpoints are proportion of patients at month 4 with more than 50% reduction in seizure frequency compared to reported seizure frequency at randomization and microfilarial load measured in skin snip. The seizure frequency data will be collected starting from day 1 using a seizure diary. Reduction of seizures will be compared between ivermectin and nonivermectin arms.

### Baseline and Follow-Up Procedures

At baseline, information will be collected on seizure semiology, frequency, risk factors, treatment history, and ivermectin treatment in the past. A full physical and neurological examination will be performed together with serological testing and skin snip examination. Weight and height measurements will be carried out and the participant body mass index will be calculated at baseline and follow-up visits. Trial participants will be instructed on how to fill out a seizure calendar and record intake of AEDs. A center visit is scheduled 2 weeks after randomization to assess potential side effects of AEDs. Side effects will be recorded using a structured questionnaire inquiring about known side effects of phenobarbital, carbamazepine, or sodium valproate. To assess seizure frequency, center visits are scheduled after 2 weeks, 1, 2, 3, and 4 months. To minimize loss to follow-up, we use Global Positioning System coordinates to locate study participants. During these visits, neurological and physical examination will be repeated, adverse events will be evaluated, and we will count AED pills for indirect measurement of AED compliance. At the fourth visit, skin snip examination and *O volvulus* serology will be repeated. Additionally, AED blood levels will be measured to directly assess AED compliance (Table 1).

**Table 1:** Overview of study baseline and follow-up assessments.

Overview procedures	Trial stage						
	t=–1	t=0	t=2w	t=m1	t=m2	t=m3	t=m4
Check of inclusion and exclusion criteria	X						
Patient characteristics questionnaire	X						
Physical exam and neurological assesment	X		X		X	X	X
Seizure diary review	X		X	X	X	X	X
Administering AED^a^/ivermectin		X	X	X	X	X	X
AED pill count	X		X	X	X	X	X
Blood exam, skin snip, urine test	X						X
Adverse event questionnaire		X	X	X	X	X	X

^a^AED: antiepilepsy drug.

If a participant is not able to visit the study center, a home visit will be performed to monitor AED use and seizure frequency.

### Sample Size Calculation

We expect that 4 months of treatment with AEDs (phenobarbital, carbamazepine, or sodium valproate) will lead to seizure freedom in 50% of the patients (experience of R Idro in Uganda). In a clinical trial performed in Rethy (Ituri) comparing the safety and parasitological efficacy of moxidectin versus ivermectin treatment in persons with *O volvulus* infection not receiving antiepileptic treatment, 6 (80%) of 7 trial participants with epilepsy were seizure free at 4 months.

Null hypothesis: The probability of seizure freedom at 4 months for immediate ivermectin treatment is equal to the probability of seizure freedom at 4 months for delayed ivermectin treatment. If we expect that seizure freedom at 4 months will be obtained in 50% of the participants with phenobarbital alone and that with additional ivermectin treatment 80% of patients will achieve seizure freedom at 4 months, about 104 subjects (52 per group) are needed to achieve the power of 90% to reject the null hypothesis at the 5% significance level. Considering that 5% of the patients will be lost to follow-up, 110 patients will be enrolled in the trial.

All comparative analyses will be based on the intention-to-treat principle: all randomized patients will be included in the analysis according to the result of the randomization.

For the primary endpoint, the null hypothesis will be tested by comparing the observed proportion of responses in arm A with the corresponding proportion in arm B at the 1-sided 5% significance level by using the Cochran-Mantel-Haenszel test for comparison of 2 independent proportions. The Cochran-Mantel-Haenszel test will be performed controlling for baseline frequency of seizures. The same test will be used for the secondary endpoint. Change versus baseline in skin micofilarial load at month 4 will be analyzed by means of a *t* test. Frequencies of seizures will be compared between participants with and without positive microfilaria skin snips at month 4.

Patients lost to follow-up will be regarded as nonresponders. Similarly, patients for whom the AED regimen had to be adapted because of an increasing number of seizures will be considered as nonresponders. AED treatment changes because of side effects or possible interactions with other drugs will not be considered as treatment failure.

### Data Handling and Record Keeping

All relevant clinical information will be collected on tablets. The identity and information of trial participants is kept confidential. Data will be entered in a Web-based electronic database compliant with Good Clinical Practice as defined by the International Conference on Harmonisation that is access-controlled and anonymized.

### Monitoring, Oversight, and Reporting

The trial sponsor is the University of Antwerp. The study team will undergo Good Clinical and Laboratory Practice protocol training and training in special procedures. An independent experienced clinical trial monitor will monitor the trial and report to the sponsor. The monitoring will include checking the consent procedure, clinical event reporting, compliance with protocol standard operating procedures, and treatment adherence. Data queries will be handled according to a quality management plan. A Data Safety Monitoring Board will be established to review safety but not for efficacy, as early-stopping for efficacy is not considered. All adverse study drug reactions, serious adverse events, and deaths will be reported to the sponsor.

### Ethics and Dissemination

The trial will be conducted in accordance with applicable laws and regulations including but not limited to the International Conference on Harmonisation guideline for Good Clinical Practice and ethical principles that have their origins in the Declaration of Helsinki. The purpose and nature of the investigation were explained to participants or parents/guardians, including risks and benefits of each procedure. If a subject wishes to participate in the study, the subject and (if applicable) parent/legal guardian must participate in the informed consent process and an informed consent/assent form must be signed or thumb-printed and dated by the subject (and parent/legal guardian, if applicable) or by his or her legally authorized representative, a literate witness (for illiterate subjects), and by the principal investigator or designee before any protocol-required procedures were performed. Ethical approval will be obtained from the ethical committee of the School of Public Health of the University of Kinshasa in the DRC and the ethical committee of the University of Antwerp.

Study results will be discussed with all stakeholders involved. The scientific community will be reached through publication in peer-reviewed open source international scientific journals and presentations at national and international scientific symposia. We will share both the overview of the research as well as the source data, inviting others to analyze and comment on the data and create their own analysis.

In collaboration with the nongovernmental organization Malteser International, a decentralized program to treat all persons with epilepsy in the villages where trial participants are recruited will be set up. Local health care workers have already been trained by 2 neurologists, and AEDs have been ordered. Malteser international has promised a sustainable provision of AEDs after the trial. Monitoring of the AED treatment program for at least 18 months is also planned.

## Results

We expect to start inclusion of patients in August 2017, and enrollment to be completed in December 2017. First results of the trial are expected by June 2018.

## Discussion

The burden of OAE in African communities is currently being investigated but is expected to be high. It has been estimated that at least 100,000 people may be affected by OAE. Current treatment with locally available AEDs is a challenge because of factors such as availability, noncompliance because of side effects, need for regular follow-up visits, daily administration, and misconceptions about the origin of seizures that prevent patients from seeking medical attention. If ivermectin treatment, potentially in addition to AEDs, is able to lead to seizure freedom or significantly reduces seizure frequency in OAE patients, this will have major consequences for epilepsy treatment in onchocerciasis-endemic regions. Ivermectin is donated for free through the Mectizan donation program [30] and is administered annually or biannually to populations at risk for onchocerciasis. Ivermectin, however, does not kill the adult worm; it only decreases its fertility [31]. After 3 to 6 months of embryostasis, the production of microfilariae resumes [32]. Therefore its effect on the frequency of seizures may also decrease over time. If this 4-month proof-of-concept trial shows a beneficial effect of ivermectin, a trial comparing a dosing of ivermectin every 3 months compared with an annual or biannual dosing needs to be considered. In a previous study, a dosing every 3 months of ivermectin caused a 30% decrease in microfilariae production [33] and was not associated with more side effects than annual dosing of ivermectin [34]. Ultimately, we aim to design a treatment strategy for OAE that can be sustained by the community. Reducing the burden of epilepsy will have a major impact on quality of life and socioeconomic status of families with affected members in Africa.

## Abbreviations

AED: antiepilepsy drug
CSF: cerebrospinal fluid
DRC: Democratic Republic of Congo
NS: nodding syndrome
OAE: onchocerciasis-associated epilepsy
